# Structural Dissection of Viral Spike-Protein Binding of SARS-CoV-2 and SARS-CoV-1 to the Human Angiotensin-Converting Enzyme 2 (ACE2) as Cellular Receptor

**DOI:** 10.3390/biomedicines9081038

**Published:** 2021-08-18

**Authors:** Deborah Giordano, Luigi De Masi, Maria Antonia Argenio, Angelo Facchiano

**Affiliations:** 1 National Research Council (CNR), Institute of Food Sciences (ISA), via Roma 64, 83100 Avellino, Italy; deborah.giordano@isa.cnr.it (D.G.), maria.antonia.argenio@gmail.com (M.A.A.); 2 National Research Council (CNR), Institute of Biosciences and BioResources (IBBR), via Università 133, 80055 Portici, Italy

**Keywords:** SARS-CoV-1, SARS-CoV-2, COVID-19, human ACE2, viral spike-protein, Receptor-Binding Domain (RBD), binding interface

## Abstract

An outbreak by a new severe acute respiratory syndrome betacoronavirus (SARS-CoV-2) has spread CoronaVirus Disease 2019 (COVID-19) all over the world. Immediately, following studies have confirmed the human Angiotensin-Converting Enzyme 2 (ACE2) as a cellular receptor of viral Spike-Protein (Sp) that mediates the CoV-2 invasion into the pulmonary host cells. Here, we compared the molecular interactions of the viral Sp from previous SARS-CoV-1 of 2002 and SARS-CoV-2 with the host ACE2 protein by in silico analysis of the available experimental structures of Sp-ACE2 complexes. The K417 amino acid residue, located in the region of Sp Receptor-Binding Domain (RBD) of the new coronavirus SARS-CoV-2, showed to have a key role for the binding to the ACE2 N-terminal region. The R426 residue of SARS-CoV-1 Sp-RBD also plays a key role, although by interacting with the central region of the ACE2 sequence. Therefore, our study evidenced peculiarities in the interactions of the two Sp-ACE2 complexes. Our outcomes were consistent with previously reported mutagenesis studies on SARS-CoV-1 and support the idea that a new and different RBD was acquired by SARS-CoV-2. These results have interesting implications and suggest further investigations.

## 1. Introduction

At the end of 2019, an epidemic cluster of unknown etiology causing interstitial bilateral pneumonia highly transmissible by human-to-human first emerged in the city of Wuhan, Hubei province, China [1,2,3]. The etiologic agent was identified as a not known before positive-strand RNA betacoronavirus (CoV-2) by the *Coronaviridae* Study Group (CSG) of the International Committee on Taxonomy of Viruses (CSG-ICTV 2020; https://talk.ictvonline.org/, accessed on 22 July 2021). The CSG-ICTV designated it as Severe Acute Respiratory Syndrome Coronavirus 2 (SARS-CoV-2), the seventh coronavirus known to infect humans, closely related to SARS-CoV-1 of 2002 with 79.5% sequence identity of probable zoonotic origin based on phylogeny, taxonomy and established practices [4,5,6,7]. Human-to-human contacts have rapidly increased the spread of the new disease, named the 2019 CoronaVirus Disease (COVID-19), mainly transmitted by respiratory bioaerosols (Flügge’s droplets), fomites and close contacts [8]. The infection rapidly spread across countries and on 2 March 2020 the World Health Organization (WHO) declared pandemic status after the infection spread out all over the world. Although there is evidence of asymptomatic and paucisymptomatic patients, forming the main infection reservoirs, severe cases of COVID-19 can evolve towards a lethal respiratory syndrome (i.e., SARS). This resulted in an extremely variable death rate, definitely lower than the coronavirus SARS-CoV-1 of 2002, but still being a serious danger to all mankind [1,9,10]. The variability observed in clinical severity would suggest a key role of the interactions between host genetic makeup and viral variants for the successful coronavirus infection.

Different human proteins act as receptors/interactors in coronavirus infection, with consequences in comorbidities [11]. Several studies on SARS-CoV-2 infectivity showed that Angiotensin-Converting Enzyme 2 (ACE2, EC 3.4.17.23), located on the host cell surface, is a protein receptor for this novel coronavirus as well as for SARS-CoV-1, which emerged in 2002 [12,13,14,15,16,17,18,19,20,21]. The viral Spike trimeric glycoprotein (Sp), anchored in the virion envelope (from which the name of crown, *corona* in Latin), mediates ACE2 receptor recognition throughout the Receptor-Binding Domain (RBD; 318–510 sequence region) of one of the three protein chains, rotated up in a receptor-accessible conformation. The amino acid sequence of this Sp portion plays a key role in conferring the ability to infect humans, being recognized as responsible for the species-specificity [5,9,22]. Sp exploits ACE2 for host cell infection, directly binding its RBD to the Peptidase Domain (PD) of receptor ACE2. Following interaction, Sp is cleaved by serine proteases, such as transmembrane protease serine 2 (TMPRSS2), into two subunits during infection: the N-terminal S1 fragment containing the RBD bound to ACE2-PD, and the C-terminal S2 fragment responsible for the membrane fusion of the virus with the host cell [13]. As a consequence, the direct interaction between Sp-RBD and ACE2-PD is the prerequisite for the invasion of human host cells by SARS-CoV-2. S1 of SARS-CoV-2 is a variable portion having around 70% amino acid identity with the corresponding sequence from SARS-CoV-1, while S2 shares 99% identity with the corresponding sequence from SARS-CoV-1 [4]. In RBD, high identity is observed except for its C-terminal region, which is involved in the direct interaction with the ACE2 receptor [4]. Consequently, to evade host immune response Sp is under selective pressure, this might explain the observed recurrent mutations in RBD. Thus, antiviral Ab against the core domain of RBD and S2 fragment could be with broad spectrum and potentially effective against viral genetic variants [23]. Additionally, Sp has been previously identified as a recombination hotspot [4,24]. Novel Sp may likely increase the virulence by evading the host immunity within species and enable host-switches by altering the cross-species ACE2 receptor recognition. Therefore, it is very likely that the amino acid sequence variability of Sp, as well as of ACE2 receptor, may modulate virion intake and consequent disease severity, making Sp a key target for vaccines, potential therapies and diagnostics [9,23]. More importantly, the RBD interface represents a robust model for predictions on structure–function relationships between accumulating mutations, virus–cell interactions, and neutralizing antibodies [9,15,23].

SARS-CoV-2 has actually resulted in many more infection cases being spread more rapidly than SARS-CoV-1. Despite the many studies carried out and in progress, it is not yet clear whether the interaction between Sp and ACE2 is stronger in SARS-CoV-1 or SARS-CoV-2, and how this is related to the different infectivity. The studies reported in the literature seem to give opposite results. Preliminary computational analysis predicted the binding free energy values between the Sp-RBD of SARS-CoV-1/CoV-2 and human ACE2, showing that the docking between RBD of SARS-CoV-1 and human ACE2 was energetically favored with respect to SARS-CoV-2 [6]. On the contrary, further modeling and molecular docking studies showed the Sp of SARS-CoV-2 had a lower binding free energy than that of SARS-CoV-1 [5]. At the same way, biophysical and structural data evidenced that the SARS-CoV-2 Sp binds to ACE2 with higher affinity (10- to 20-fold) than Sp of SARS-CoV-1 on the basis of the cryo–electron microscopy structure of the SARS-CoV-2 Sp in the prefusion conformation and kinetics quantification of their interaction by surface plasmon resonance [20]. The same study has evidenced many distinctions at molecular level when Sp of SARS-CoV-1 and SARS-CoV-2 interact with ACE2.

SARS-CoV-2 uses the ACE2 receptor more efficiently than SARS-CoV-1 strain of 2003, but less efficiently than SARS-CoV-1 strain of 2002 [18,22]. SARS-CoV-2 mutations located into the Sp-RBD likely determine higher infectivity and lower pathogenicity than SARS-CoV-1 of 2002 (around 10% mortality rate) [15,18,24]. Although the genome of SARS-CoV-2 has 82% nucleotide identity with SARS-CoV-1 and the Sp of SARS-CoV-2 shares about 76% sequence identity with that of SARS-CoV-1 [4], Othman et al. recently showed the binding free energy scores with human ACE2 are similar between SARS-CoV-2 and SARS-CoV-1 [17]. The same results were consistent with the fact that the interface amino acid residues of the SARS-CoV-2 RBD might have evolved in a complex way from progenitor strains rather than accumulating mutations or to be the result of artificial manipulations by genetic engineering. All together, these studies disagree on how the structural differences in the binding of Sp to ACE2 between the two coronaviruses can stabilize or destabilize the ligand-receptor complex of Sp-ACE2.

Moreover, since there are no specific therapeutic agents, and several vaccines are currently available, a deeper understanding of the molecular mechanisms underlying the initial steps of infection is required to reveal further potential therapeutic targets. First interventions were directed against the SARS-CoV-2 that emerged in 2019, but the recent emergence of new SARS-CoV-2 genetic variants, also related to mutations in the Sp, presents new challenges for their high transmissibility and for putting in doubt the efficacy of current vaccines. Therefore, we in silico investigated the structural features and the surface interactions at the chain-chain interface in Sp-ACE2 complexes of available crystallographic structures of both SARS-CoV-1 and CoV-2. The results showed what amino acid residues are involved and how they interact at the binding interface, at the same time thus evidencing interesting structural characteristics and differences between SARS-CoV-1 and CoV-2. Therapy and prevention of COVID-19 could benefit from the present study, because Sp is a potential target for drug design and a viral antigen for optimal vaccine and monoclonal antibody development. Potential implications on the health impact of the most recent coronavirus are discussed.

## 2. Materials and Methods

To analyze the amino acids located at the interface of the bond between ACE2 and the RBD of Sp from SARS-CoV-1 and SARS-CoV-2, the structures annotated as ACE2-RBD complex available in Protein Data Bank (PDB) (https://www.rcsb.org/, accessed on 22 July 2021) have been analyzed. In the case of Sp from SARS-CoV-1, several structures complexed with ACE2 are available and a selection has been made by considering experimental method, resolution, qualitative parameters (R-free, R-work, general PDB validation plot), missing atoms or missing residues, being all these information available in the PDB file. Moreover, Z-score and Ramachandran plot have been considered, as determined by ProSA-web [25] and PROCHECK [26], respectively. Four structures were judged suitable in terms of completeness and structural quality for the analysis of complex formed between ACE2 and Sp of SARS-CoV-1, i.e., 2AJF (two chain interfaces: A:E and B:F), 6CS2, 6ACJ, 6ACK [15,16,27]. For Sp from SARS-CoV-2, three structures of complexes with ACE2 were available in PDB, i.e., 6LZG (one chain interface: A:B), 6M0J (one chain interface: A:E), 6M17 (two chain interfaces: B:E and D:F) and, after a similar evaluation, all were selected for the analysis [19,21,28]. To detect amino acid residues responsible for the interface contacts in each complex, we used different criteria at decreasing priority, similarly to our previous studies [29]. The higher priority was assigned to H-bonds and salt bridges recognized between the two protein chains, followed by generic interactions not recognized as H-bonds or salt bridges, but possible at distance between atoms of the two protein chains lower than a threshold value, here fixed at 6 Angstroms, as lower energy interactions. The search of the interface contacts was performed by PyMOL 1.3 (https://pymol.org/, accessed on 22 July 2021) used as local installation, and by PDBePISA (Proteins, Interfaces, Structures and Assemblies) service at the European Bioinformatics Institute (http://www.ebi.ac.uk/pdbe/prot_int/pistart.html, accessed on 22 July 2021) by choosing the x,y,z symmetry operation [30], which recognized the residues involved in H-bond or salt-bridge. Furthermore, COCOMAPS [31] has been used to determine the proximity of amino acids at the inter-chain surface, by using the cut-off distance of 6 Angstroms.

## 3. Results and Discussion

### 3.1. Comparison of the S-Protein Sequences and Amino Acids from SARS-CoV-1 and SARS-CoV-2 at Sp-ACE2 Complex Interfaces

**Figure 1:**
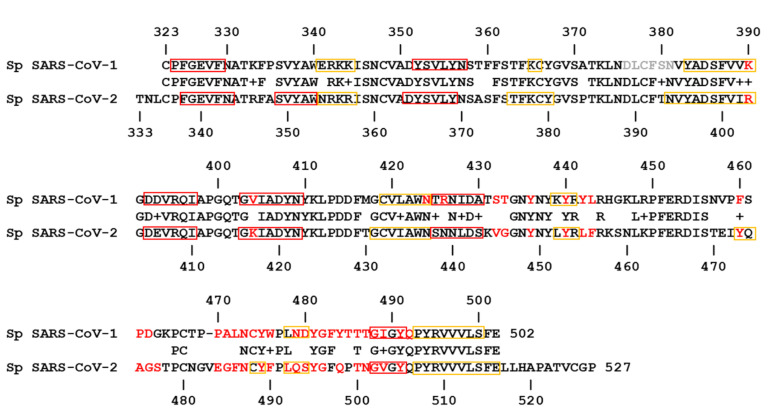
Sequence alignment of Sp receptor binding domains (RBD) from SARS-CoV-1 and SARS-CoV-2. The amino acids that interact with hACE2 at least in one of the complex structures are in red. The consensus sequence between the two coronavirus sequences reports the conserved amino acids and the “+” symbol when the amino acid is different but similar. Grey letters indicate amino acids of SARS-CoV-1 for which the 3D structure is not determined. Secondary structure elements are evidenced by boxes (red for alpha helices, yellow for beta strands).

The amino acid sequences of Sp receptor binding domains (RBDs) from SARS-CoV-1 and SARS-CoV-2 are very similar, sharing approximately 60% of identical amino acids (Figure 1). As it is visible in Figure 1, most of the sequence differences between SARS-CoV-1 and SARS-CoV-2 are present in two regions of RBD, i.e., from position 430 to 462 and from position 470 to 503 (CoV-2 numbering). To understand the specific molecular interactions underlying the binding of Sp to ACE2, we investigated in silico the X-ray crystallographic structures, available in the public domain Protein Data Bank (PDB), of human ACE2 in complex with SARS-CoV-2/CoV-1 Sp, by analyzing in detail the interacting amino acids at the chain-chain interfaces. The complete network of interactions between the Sp and ACE2, in each complex for both SARS-CoV-1 and SARS-CoV-2, is reported in detail in Supplementary Materials, Table S1. There were respectively 29 and 33 amino acid residues of Sp from SARS-CoV-2 and SARS-CoV-1 (Figure 1) that interact with 32 and 33, respectively, amino acid residues of receptor ACE2 at least in one of the complex structures (Table S1). It is interesting to note that most of the Sp amino acids involved in the binding interface are located right in correspondence of the two RBD regions differing between the two Sp. In more detail, out of 29 interacting amino acids of SARS-CoV-2 Sp, 23 residues interact with the receptor in all investigated complexes, as reported in Table 1, i.e., under different experimental conditions of protein crystal formation. Therefore, their interactions may be particularly relevant for the virus-specific recognition modes of the ACE2 protein, acting as a viral receptor on the surface of the plasma membrane of the target cells. The other six residues of SARS-CoV-2 Sp interact with ACE2 only in a few complex structures, likely due to the differences in the experimental conditions of protein crystal formation: R403, S477, E484, L492, S494, Y495 (Table S1). Consequently, the interactions of some of these last amino acids might be less relevant as they were observed only in a few cases. Similarly, out of 33 amino acids of SARS-CoV-1 Sp, 23 residues interact with ACE2 in all investigated complexes, as reported in Table 1. The other ten residues of SARS-CoV-1 Sp interact with ACE2 only in a few complex structures: K390, V404, N424, T433, P470, A471, C474, W476, D480, F483 (Table S1).

By comparing the two lists of 23 amino acids from SARS-CoV-2 and SARS-CoV-1, we can identify 19 residues that have a correspondence in the two aligned Sp sequences for all analyzed complexes (see Figure 1 and Table 1a,b). Out of these, eight amino acid residues are identical in both sequences and are grouped in Table 1a. Another identical residue interacts with ACE2 in all complexes with SARS-CoV-1 Sp, i.e., Y481, and in all but one with SARS-CoV-2 Sp, i.e., Y495 (Table S1). The identical amino acids with interactions confirmed in all complexes may have a key function in the binding of SARS-CoV-2/CoV-1 Sp with the receptor ACE2 on the target cell. Although they are located in the two differing binding regions, evidently important structural and/or functional constraints may have protected these residues from evolutionary pressure. Accordingly, they can contribute to identifying the main conserved Sp epitopes available for antibody elicitation upon vaccination, to be exploited as effective targets also in case of Sp variants [23,32].

Nevertheless, the other 15 amino acids are also notable, being specific to each coronavirus Sp and involved in the interactions of all available complexes Sp formed with ACE2. Out of 15 residues, in Table 1b we listed the 11 different Sp amino acids of both coronaviruses that correspond at the same position in the sequence alignment as reported in Figure 1. In some cases, the two Sp have amino acids with similar properties (i.e., Y473/F460, Q493/N479, N501/T487, V503/I489) that interact similarly with ACE2 (see for details Table S1). In the other cases, most of the interactions are similar, with some additional interaction in case of larger side chains. In particular, in the cases of F456/L443 and F486/L472 we noted that the two phenylalanines of SARS-CoV-2, differently from leucines of SARS-CoV-1, may form additional aromatic interactions with F28 and Y83 of ACE2, respectively (see for details Table S1). However, the different amino acid residues at the same sequence positions in the two coronaviruses (Table 1b) and, at the same time, the conservation for most of their chemical interactions with ACE2 could play a role similar to that of the identical residues of Table 1a in the formation and stability of Sp-ACE2 prefusion complex.

Finally, Table 1c lists the amino acids that interact in all Sp-ACE2 complexes for only one of the two coronaviruses. The four peculiar residues of SARS-CoV-2 Sp i.e., K417, G446, G485, and F490, correspond in the sequence alignment (Figure 1) to residues of SARS-CoV-1 Sp that interact in one (A471) or two (V404, T433, W476) complexes (Table S1). We note that in the case of glycines the interactions are evidently due to the backbone moiety, and in the case of F490/W476 H-bond or generic interaction occurs with K31 of ACE2. The most peculiar interaction observed is related to the residue K417, located before the first variable region of the RBD, that strongly binds to D30 of ACE2, as the interatomic distance and orientation allows to form H-bond and salt bridge. Therefore, the interaction K417-D30 plays an important structural difference in the binding to ACE2 of the SARS-CoV-2 Sp in comparison to the Sp of SARS-CoV-1. On the other side, concerning the four peculiar residues of SARS-CoV-1 Sp that interact with ACE2 in all complexes, the above mentioned Y481 forming a H-bond with K353 corresponds to Y495 in SARS-CoV-2 Sp, binding ACE2 in most of the complexes, while R426, T485 and Q492 correspond to SARS-CoV-2 residues that do not interact with ACE2 at all, i.e., N439, P499, and Q506, respectively. In the case of T485 and Q492, we note that their atomic contacts make weak bounds at the surface (see for details Table S1). For the residue R426, located in the first variable region of RBD, there are the suitable geometrical conditions to form H-bond with Q325, and both H-bond and salt bridge (HS) with E329 (see for details Table S1). This observation highlights that R426 may determine the most important structural difference of SARS-CoV-1 Sp in the binding to ACE2. The discriminative residues K417 and R426, located in the alignment of Figure 1 at a distance of 22 residues between the two coronaviruses, likely indicate that ACE2 can utilize very different molecular interactions to bind the Sp of SARS-CoV-1 and SARS-CoV-2.

### 3.2. Different Regions of ACE2 Are Involved in the Interaction with Sp of SARS-CoV-1 and SARS-CoV-2

To investigate the counterpart ACE2 of the SARS-CoV-2/CoV-1 Sp in the same experimental complexes, we have also analyzed in detail the amino acid residues of ACE2. The network of interactions occurring between ACE2 and the Sp, only for pairs of amino acids interacting in all complexes, is shown in Figure S1 for both coronaviruses. As already reported above, we found 32 ACE2 amino acid residues interacting with SARS-CoV-2 and 33 with SARS-CoV-1, respectively, and 30 of them are in common (Supplementary Figure S1). The two ACE2 residues I21 and A36 interact exclusively with the Sp of SARS-CoV-2 by making few weak interactions with G476 and Q493, respectively. The three ACE2 residues Q76, T78, E329 interact exclusively with Y475, L472, and T485/T486/T487/Q492, respectively, of the Sp of SARS-CoV-1 also forming generic interactions, with the important exception of the couple E329-R426 with salt bridge and H-bond, as already discussed above.

**Figure 2:**
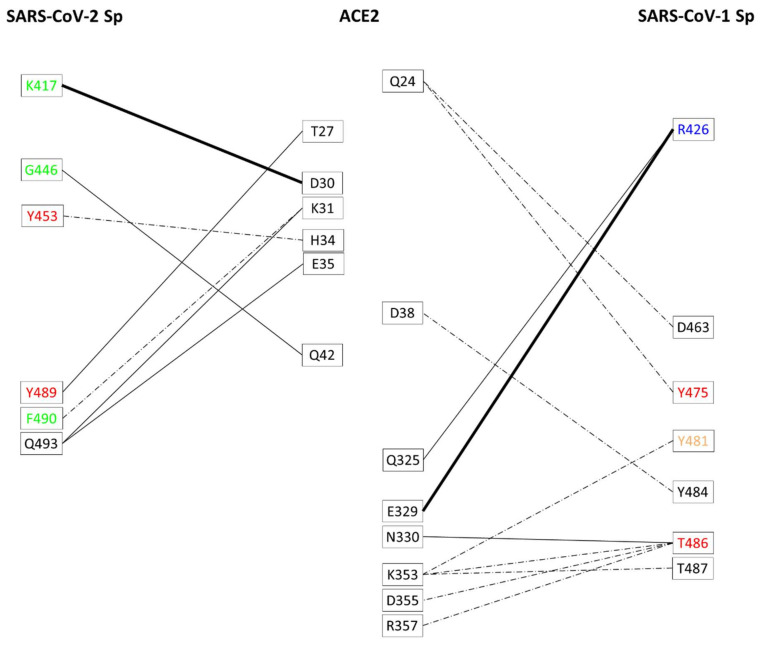
Network of interactions between ACE and Sp, restricted to only different H-bond and salt-bridge interactions. The networks of ACE2 with Sp of SARS-CoV-1 and SARS-CoV-2 are presented side-by-side, with ACE2 residues horizontally aligned by their sequence number, and Sp residues horizontally aligned by their alignment in Figure 1. Large lines indicate salt bridges (always observed with also H-bonds), thin lines indicate H-bonds. Dashed lines indicate interactions observed in less than half of the complexes. Sp amino acids are colored following the code: red = amino acids listed in Table 1a; black = amino acids listed in Table 1b; green and blue = amino acids listed in Table 1c (for SARS-CoV-2 and CoV-1, respectively); orange = amino acid of Sp SARS-CoV-1 also listed in Table 1c with a peculiar feature (see Table 1 note).

Both the Sp interact widely with two sequence regions at the surface of ACE2, i.e., an N-terminal region comprising residues from 19 to 83, and a central sequence region comprising residues from 324 to 393 (Figure S1). Although the network is very complex, it is particularly interesting to note that both coronaviruses bind the two regions of ACE2, and the schematic representation puts in evidence some peculiarities. To simplify the view and investigate in more detail the network, we showed in Figure 2 only the H-bond and salt bridge interactions that differ between the two coronaviruses, as these interactions are energetically more relevant than the other generic interactions detected (see Section 2, Materials and Methods for details). These differences evidence that the SARS-CoV-1 Sp has more discriminative interactions with the ACE2 central region (324–393) than with the N-terminal region (19–83). These bonds include the strong interactions of Sp R426 with ACE2 Q325 and E329, already evidenced above as an important structural difference. Moreover, we found differences in interactions of ACE2 N330 with Sp T486 in all complexes, while for K353, D355, and R357 only in some of the complexes. Then, K353 of ACE2 forms discriminative H-bonds with Y481/T486/T487. Q24 and D38 of ACE2 are the unique residues of N-terminal region (19–83) that form discriminative H-bonds with D463/Y475 and Y484, respectively, of Sp of SARS-CoV-1 in some of the complexes. It is interesting to note that the region of ACE2 with more interactions with Sp of SARS-CoV-1 includes also the residues responsible of substrate binding, i.e., 345–346 [33]. At the opposite, SARS-CoV-2 Sp forms discriminative interactions only with the N-terminal region (19–83) of ACE2, and in this case a salt bridge is formed between Sp K417 and ACE2 D30 (Figure 2), already indicated above as important structural difference. Moreover, other H-bond interactions are detected for ACE2 T27 with Sp Y489, K31 with F490 and Q493, H34 with Y453, E35 with Q493, and Q42 with G446. It is interesting to note that K417, G446, and F490 are reported in Table 1c as they interact with ACE2 in all complexes, differently from the corresponding residues in SARS-CoV-1. These different residue-residue interactions suggest that the two Sp, while interacting with the receptor ACE2 at the same surface regions (Figure S1), are anchored with different strength to the two different districts of the surface (Figure 2). This observation could evidence differences at the level of the molecular mechanism of cell infection and virulence, also in light of recent findings. ACE2 has a negatively charged binding surface while Sp-RBDs are overall positively charged, forming the complex Sp-ACE2 by attractive forces. At the same time, different amino acid residues and salt bridge/H-bond positions utilized by Sp of SARS-CoV-1 and SARS-CoV-2 allow them to bind differently with human ACE2 [34]. This is also in agreement with the observation that the RBD is recognized mainly by polar amino acid residues located into the extracellular PD of ACE2 [20,21]. Actually, amino acid differences occurring in the two coronaviruses could have consequences on their infection capability by modulating the interaction on the two surface regions of ACE2 we identified as discriminative for each Sp.

### 3.3. Predictions on Impact of Coronavirus Variants at the Sp-ACE2 Binding Interface

The modulation of the interaction on the two surface regions of ACE2 could be related to the different infection capability also for the SARS-CoV-2 variants. Our structural comparison of the amino acids at the interface could be exploited to design solutions effective against the binding to the receptor ACE2 of the SARS-CoV-2 Sp and its mutational variants created under natural selection conditions. To date, four major lineages with variants of potential biological significance were identified (https://cov-lineages.org; https://covariants.org, accessed on 22 July 2021), by focusing only on the variations affecting the Sp-RBD: Alpha (B.1.1.7) with the mutation N501Y; Beta (B.1.351) and Gamma (P.1) with the same three mutations K417N, E484K, and N501Y (also in Alpha); and Delta (B.1.617) with the two mutations L452R and T478K. Among these amino acid residues underwent natural mutational events, in our study, K417 was found to be extremely critical in the binding of Sp to ACE2. K417 forms salt bridge and H-bond interactions with D30 of ACE2, while the K417N substitution might determine a weaker bond for the Beta and Gamma variants, by loss of the positive charge of lysine. On the contrary, N501 interacts with Y41 of ACE2 (as T487 of SARS-CoV-1 Sp) by an H-bond, and the N501Y substitution of Alpha, Beta, and Gamma variants, by introducing a tyrosine side chain, might maintain the H-bond and add an aromatic interaction with Y41, thus making stronger the binding to ACE2. However, the sterical hindrance of the tyrosine might introduce perturbation at the interface. In fact, very recently Socher et al. [35] performed molecular dynamics simulations also on this substitution showing a reduced binding affinity of B.1.1.7 Sp for ACE2, as the residue Y501 de-restructured the Sp-ACE2 interface decreasing the linear interaction energy between the RBD and ACE2.

It is interesting to note that the other Sp mutations, i.e., E484K, L452R, and T478K, could have been selected as they affect amino acids that are not involved in significant interactions in wt SARS-CoV-2. Importantly, the three mutations introduce positive charges on Sp, with a possible gain of charge-charge interaction on the ACE2 surface, in agreement with the investigations by Xie et al. [34], already mentioned in the previous paragraph. In the case of E484, in fact, we note that this amino acid is not involved in relevant interactions with ACE2 K31, while the corresponding amino acid in SARS-CoV-1, i.e., P470, is capable of generic interaction with ACE2 E75 (see Table S1). Substitutions with positive charges on Sp may create further positive-negative charge interactions that make possible an increased binding affinity of Sp to the ACE2 receptor. The close spatial proximity of D30-K417 and K31-E484 opposite-charge couples suggests that the double mutation observed in Beta and Gamma variants (i.e., K417N and E484K) removed the two interactions, but maintained in the region at least the positive lysine side chain on the SARS-CoV-2 surface, which might preserve in that region at least one opposite-charge couple. However, the molecular impact of mutations within RBD on Sp binding stability with ACE2 is yet largely unknown and needs further investigations [35].

### 3.4. Molecular Aspects of the Peculiar Interactions at the Sp-ACE2 Binding Interface

**Figure 3:**
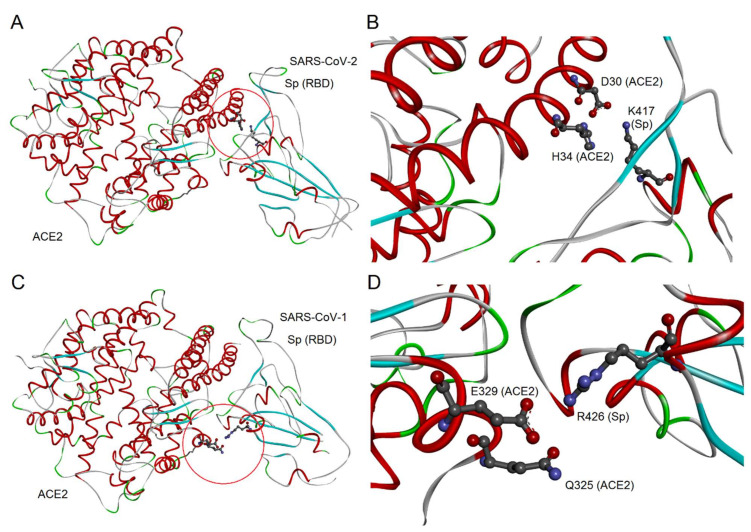
Molecular view of peculiar interactions. (**A**) The interaction of ACE2 with Sp of SARS-Cov-2, both proteins represented as backbone ribbon, with helix regions in red, beta-strand regions in cyan, and turns in green. The red circle indicates the region with Sp-K417. (**B**) The detailed view of the interactions of Sp-K417 with ACE2 D30 and H34. (**C**) The interaction of ACE2 with Sp of SARS-CoV-1, with backbone representation and colors as in (**A**). The circle indicates the region with Sp-R426. (**D**) The detailed view of the interactions of Sp-R426 with ACE2 Q325 and E329.

As already stated above, a significant role in the Sp-ACE2 interaction can be played by the positively charged K417 of SARS-CoV-2 Sp, that corresponds in SARS-CoV-1 Sp to the apolar residue V404 (Table S1). Indeed, seeing that the geometrical conditions for salt bridge as well as H-bond formation are satisfied, the positive charge on the K417 side chain interacts with the negative charge of the D30 side chain and generic interactions are made with H34 on the ACE2 surface (Figure 3A,B). Evidently, the salt bridge and H-bond interactions are not possible for the SARS-CoV-1 Sp amino acid V404 corresponding in the sequence alignment (Figure 1). On the other side, also in the case of SARS-CoV-1 Sp interaction with ACE2 we note a residue, i.e., the positively charged R426 that interacts in almost all Sp-ACE2 complexes with a negatively charged E329 residue of ACE2 surface, without a corresponding interaction for SARS-CoV-2 (Figure 3C,D). As reported above, two different regions of ACE2 are involved in salt bridges with Sp, as it is visible by comparison of Figure 3A, for the interaction with SARS-CoV-2 in the N-terminal region of ACE2, and Figure 3C, mainly for the interaction with SARS-CoV-1 in the central region of ACE2.

Moreover, the negatively charged residue E484 of SARS-CoV-2 Sp (mutated in K484 in the Beta and Gamma variants), that in the sequence alignment (Figure 1) corresponds to the apolar residue P470 of SARS-CoV-1 Sp, interacts with K31 on ACE2 surface in two out four Sp-ACE2 complexes (Table S1). Due to the close proximity of D30-K417 and K31-E484 (the pair of opposite-charge couples discussed for the variant mutations), we may suppose that the location of these two positive-negative charge couplings may represent a surface site of strong binding interaction between Sp and ACE2 and a unique feature of the SARS-CoV-2 Sp.

Another interesting observation concerns the interaction differences in residue pairs with aromatic side chains. Amino acid F486 of SARS-CoV-2 Sp interacts with Y83 of ACE2 by no relevant interactions together with other amino acids (Table S1). In SARS-CoV-1, the corresponding amino acid L472 also interacts with Y83 of ACE2, together with other amino acids. However, we note that the stacking interaction between the two aromatic side chains is possible only in the case of SARS-CoV-2 (i.e., Sp F486 and ACE2 Y83). Similarly, additional stacking interactions between the aromatic side chains of SARS-CoV-2 F456 and ACE2 F28 are also possible (Table S1), whereas the corresponding SARS-CoV-1 L443 does not interact with aromatic side chains of ACE2. Both cases might suggest that SARS-CoV-2 Sp interaction with ACE2 may be characterized by more aromatic interactions than SARS-CoV-1 Sp. However, also SARS-CoV-1 Y484 interacts with an aromatic side chain ACE2, i.e., Y41, and the corresponding SARS-CoV-2 Q498 cannot create stacking interaction with aromatic side chains of ACE2. Altogether, the aromatic interactions at the interface appear as an interesting feature that contribute to stabilizing the complex state by burying the hydrophobic rings, and at the same time to different modes of interaction of the two viruses with ACE2. Our in silico analysis showed interesting structural characteristics and differences between Sp of SARS-CoV-1 and CoV-2 based on discriminative amino acid residues at the virus-cell receptor binding interface. Since Sp of CoV-2 represents an optimal target for drug design and vaccine and monoclonal antibody development, then therapy and prevention of COVID-19 could potentially benefit from this study.

### 3.5. Comparison with Mutagenesis Studies on Sp and ACE2 from the Previous SARS Outbreaks

A previous research compared Sp from two very different human outbreaks and from palm-civets implicated in the transmission of SARS-CoV-1 to humans [15]. The affinity of Sp for ACE2 was determinant in the viral infection and in the severity of disease. Sp from the severe 2002 outbreak efficiently bound human ACE2 (hACE), while Sp from the mild 2003 outbreak and palm-civets bound hACE2 with lower affinity. The low-affinity association of Sp from palm-civets to hACE2 was complemented by mutagenesis of the amino acids residues K479N and S487T of the earlier severe outbreak. To confirm this, the double mutation N479/T487 → K479/S487, from human coronavirus to its palm-civet counterpart, strongly inhibited interaction with hACE2. The presence of K479 in Sp from the virus of palm-civet and of the residue K31 in human ACE2 shows that these two positively charged residues of lysine facing each other repulsively interfere with the formation of the complex Sp-hACE2. The repulsive effect is not present in the palm civet infection, because in palm-civet ACE2 there is T31. The two residues N479 and T487 correspond respectively to the SARS-CoV-2 residues Q493 and N501 identified in the present study as interacting with hACE2 in all available experimental complexes (Table 1b). Our in silico results confirmed previous experimental findings on SARS-CoV-1, allowing to hypothesize a key role of the two residues Q493 and N501 in the SARS-CoV-2 high-affinity binding to hACE2. In fact, our in silico data show that Q493 stably interacts with the residue K31 and H35 (Table S1, Figure 2). Additionally, residue K31 interacts with the residue E484 and the two residues F490 and L492 upstream of Q493. At the same way, T487 is conserved in the Sp from the severe 2002 outbreak, but this residue is not present in the Sp from the mild 2003 outbreak and palm-civet virus that have S487, while it corresponds to the residue N501 of SARS-CoV-2. Mutagenesis analysis of position 487 showed the key role of a threonine in increasing the affinity for human and non-human ACE2, essentially for the presence of an additional methyl group in the side chain of this amino acid, while the terminal hydroxyl group is conserved. To confirm this, our in silico analysis shows that N501 stably interacts with the residue Y41 of hACE2 (also present in the palm-civet and rat ACE2), which in turn further interacts with the residues Q498 and T500 upstream of N501 (Table S1).

These observations collectively suggest that Sp alterations in the positions 493 and 501 (479 and 487 of SARS-CoV-1) may be sufficient for the adaptation of SARS-CoV-2 to the human cells accommodating the K31 and Y41 residues of ACE2 receptor. Consequently, the presence of these or other residues in these mutation hotspot regions may be an important element predictive in the risk assessment of new SARS-CoV-2 variants/lineages [36]. Finally, in our analysis, we evidenced the presence of important interactions by R426 of the Sp from SARS-CoV-1, not subject to mutagenesis in that study [15], as well as K417 of Sp from SARS-Cov-2, corresponding to V404 of Sp from SARS-CoV-1, obviously not yet known at that time.

In the same study and with a similar approach, Li et al. [15] complemented the lower affinity of Sp from palm-civet by altering hACE2 to the palm-civet counterpart in the specific amino acid residues 31–40 and K353. Furthermore, the SARS-CoV-1 Sp-binding domain on hACE2 was localized by characterizing the human-rat ACE2 chimeras [15]. In addition, the alterations to their hACE2 counterpart of the amino acids NFS → MYP (residues 82–84) and H353K converts rat ACE2, which does not bind SARS-CoV-1 Sp, to an efficient receptor. The alterations of the two residues K31D and Y41A on the first helix of human ACE2 interfered with SARS-CoV-1 Sp binding, as also shown by altering the residues D355A and R357A, both downstream of K353 on ACE2. All together, these key residues mapped the Sp-binding site on ACE2 above the deep cleft that harbors the active site [15]. The results presented by Li et al. [15] are confirmed by our in silico analysis of experimental complexes and can be at least partially applied to the interaction of the Sp of SARS-CoV-2. In fact, we evidenced an interacting region of hACE2 from 19 to 45 (Figure S1), even more interacting with Sp of SARS-Cov-2 (Figure 2). Furthermore, other shorter regions with important interactions are found for hACE2 in 76–83, thus including Y83, in 324–330, and in 352–357, including K353, D355, and R357 (Figure S1). However, these latter three amino acids are in our analysis more important for the interaction with Sp of SARS-CoV-1 than SARS-CoV-2 (Figure 2). Moreover, in our analysis, we evidenced the interactions of Q325 (H-bond) and E329 (H-bond and salt bridge) with R426 of the Sp from SARS-CoV-1. In spite of that, the mutagenesis study on Q325P and E329T to rat ACE2 (in combination with N322Q) did not show a significant effect on the interaction of SARS-CoV-1 Sp with ACE2 [15]. As shown in Figure 2, Sp of SARS-CoV-1 has many more interactions than SARS-CoV-2 with the hACE region from 325 to 357, these can counterbalance the loss of interactions by mutagenesis and explain why they have no effect.

## 4. Conclusions

The molecular interactions in the Sp-ACE2 complex are of crucial importance for the successful coronavirus attack, and their complete understanding can be useful for the definition of the mechanism of action as well as for ascertaining possible interaction differences in the case of Sp variants. Differences in the interaction of Sp from SARS-CoV-1 and CoV-2 with ACE2 may be responsible for the higher spread of the infection started in 2019, in comparison to the 2002. More recent studies propose that higher affinity of SARS-CoV-2 than SARS-CoV-1 Sp for human ACE2 may contribute to the ease with which SARS-CoV-2 can spread from human to human. Our structural analysis on the crystallographic complexes of Sp-ACE2 define what amino acid residues interact at the chain-chain interface and how they interact at molecular level, evidencing interesting differences and structural characteristics peculiar to SARS-CoV-1 and CoV-2 with potential implications on the different health impact of the most recent coronavirus. Our study evidences that Sp from SARS-CoV-2 binds to the hACE2 cellular receptor by interacting on the surface regions differently from Sp of SARS-CoV-1, and this might be related to differences in the cell infectivity, regardless of their higher or lower binding affinity. We evidenced for the first time that the complex network of the interactions at the interface between ACE2 and Sp indicates stronger interaction of Sp from SARS-CoV-2 with the N-terminal portion of ACE2, while Sp from SARS-CoV-1 has more interactions with the central portion of ACE2. However, additional studies are needed to explore how our findings can be exploited for helping to unveil the molecular basis of recognition, and consequently invasion, transmission, spread, and pathogenesis of SARS-CoV-2, as well as for optimizing diagnostic, antiviral and vaccination strategies.

## Figures and Tables

**Table 1:** The 23 amino acid residues of SARS-CoV-2 and SARS-CoV-1 Sp interacting with ACE2 at the binding interface in all the crystallographic structures analyzed.

SARS-CoV-2 Sp	SARS-CoV-1 Sp
(a) 8 amino acids are identical at the corresponding positions	
Y449	Y436
Y453	Y440
N487	N473
Y489	Y475
G496	G482
T500	T486
G502	G488
Y505	Y491
(b) 11 amino acids are different at the corresponding positions	
V445	S432
L455	Y442
F456	L443
Y473	F460
A475	P462
G476	D463
F486	L472
Q493	N479
Q498	Y484
N501	T487
V503	I489
(c) 4 amino acids for each Sp have no correspondent interactions	
K417	-
-	R426
G446	-
G485	-
F490	-
-	Y481 *
-	T485
-	Q492

* Identical amino acid is present in Sp of SARS-CoV-2, i.e., Y495, and it interacts with ACE2 in three out four complexes.

## Data Availability

Data used in this study have been retrieved from public databases as described in Materials and Methods, Section 2.

## Supplementary Materials

The following are available online at https://www.mdpi.com/article/10.3390/biomedicines9081038/s1, Figure S1: Network of interactions between ACE and Sp; Table S1: Amino acid residues of SARS-CoV-2 and SARS-CoV-1 Sp interacting with ACE2 at the binding interface.
