# Supplementary material for: Structural Dissection of Viral Spike-Protein Binding of SARS-CoV-2 and SARS-CoV-1 to the Human Angiotensin-Converting Enzyme 2 (ACE2) as Cellular Receptor
Source: Biomedicines. 2021 Aug 18;9(8):1038. doi: 10.3390/biomedicines9081038 (PMC8394803; doi:10.3390/biomedicines9081038)
Supplement: Supplementary file 1 [file biomedicines-09-01038-s001.zip › Table-S1.pdf]

Table S1. Amino acid residues of SARS-CoV-2 and SARS-CoV-1 Sp interacting with ACE2 at the binding interface, and vice versa. The amino acids of ACE2 are followed by letters in parentheses to indicate the type of interaction at the surface: H-bond (H), salt bridge (S), or generic interaction (I). The PDB code of each ACE2-Sp complex is here reported, as indicated in Material and Methods section, in the case followed by letters in parentheses to indicate the two interface chains. Sp amino acids are colored following the code: red = amino acids listed in Table 1a; black = amino acids listed in Table 1b; green and blue = amino acids listed in Table 1c (for SARS-CoV-2 and CoV-1, respectively); orange = amino acid of Sp SARS-CoV-1 also listed in Table 1c with a peculiar feature (see Table 1 note); grey = amino acids not listed in Table 1 because they do not interact with ACE2 in all complexes.

**SARS-CoV-2 Sp amino acids**	**ACE2 amino acids interacting with SARS-CoV-2 Sp**				**SARS-CoV-1 Sp amino acids**	**ACE2 amino acids interacting with SARS-CoV-1 Sp**				
	**6LZG**	**6M0J**	**6M17 (B:E)**	**6M17 (D:F)**		**2AJF (A:E)**	**2AJF (B:F)**	**6CS2 (B:D)**	**6ACJ (C:D)**	**6ACK (C:D)**
R403		E37 (I)	E37 (I)	E37 (I)	K390			H34 (I)	H34 (I)	
K417	D30 (HS)<br>H34 (I)	D30 (HS)<br>H34 (I)	D30 (I)	D30 (I)	V404	H34 (I)	D30 (I)<br>H34 (I)		H34 (I)	
					N424			Q325 (I)		
					R426	Q325 (H)<br>G326 (I)<br>E329 (HS)	Q325 (I)<br>G326 (I)<br>E329 (HS)	Q325 (I)<br>G326 (I)<br>E329 (HS)	Q325 (H)	Q325 (H)<br>E329 (I)
V445	L45 (I)	L45 (I)	L45 (I)	L45 (I)	S432	L45 (I)	L45 (I)	L45 (I)	Y41 (I)<br>L45 (I)	Y41 (I)<br>L45 (I)
G446	Q42 (H)<br>L45 (I)	Q42 (H)<br>L45 (I)	Q42 (I)	Q42 (I)	T433				Q42 (I)<br>L45 (I)	Q42 (I)
Y449	D38 (H)<br>Q42 (H)	D38 (H)<br>Q42 (H)<br>K353 (I)	D38 (H)<br>Q42 (I)	D38 (H)<br>Q42 (I)	Y436	D38 (H)<br>Q42 (H)	D38 (H)<br>Q42 (H)<br>K353 (I)	D38 (H)<br>Q42 (H)<br>K353 (I)	D38 (H)<br>Y41 (I)<br>Q42 (I)<br>K353 (I)	D38 (H)<br>Y41 (I)<br>Q42 (H)<br>K353 (I)

Y453	H34 (H)	H34 (I)	H34 (I)	H34 (I)	Y440	H34 (I)	H34 (I)	H34 (I)	H34 (I)	H34 (I)
L455	D30 (I)<br>K31 (I)<br>H34 (I)	D30 (I)<br>K31 (I)<br>H34 (I)	D30 (I)<br>K31 (I)<br>H34 (I)	D30 (I)<br>K31 (I)<br>H34 (I)	Y442	T27 (I)<br>D30 (I)<br>K31 (I)<br>H34 (I)	T27 (I)<br>D30 (I)<br>K31 (I)<br>H34 (I)	T27 (I)<br>D30 (I)<br>K31 (I)<br>H34 (I)<br>E35 (I)	T27 (I)<br>D30 (I)<br>K31 (I)<br>H34 (I)<br>E35 (I)	D30 (I)<br>K31 (I)<br>H34 (I)
F456	T27 (I)<br>F28 (I)<br>D30 (I)<br>K31 (I)	T27 (I)<br>F28 (I)<br>D30 (I)<br>K31 (I)	T27 (I)<br>F28 (I)<br>D30 (I)<br>K31 (I)	T27 (I)<br>F28 (I)<br>D30 (I)<br>K31 (I)	L443	T27 (I)<br>K31 (I)	T27 (I)	T27 (I)<br>D30 (I)	T27 (I)	T27 (I)
Y473	T27 (I)	T27 (I)	T27 (I)	T27 (I)	F460	T27 (I)	T27 (I)	T27 (I)	T27 (I)	T27 (I)
A475	S19 (H)<br>T20 (I)<br>E23 (I)<br>Q24 (I)<br>T27 (I)	S19 (I)<br>Q24 (I)<br>T27 (I)	Q24 (I)<br>T27 (I)	Q24 (I)<br>T27 (I)	P462	S19 (I)<br>E23 (I)<br>Q24 (I)<br>T27 (I)	S19 (H)<br>E23 (I)<br>Q24 (I)<br>T27 (I)	S19 (H)<br>E23 (I)<br>Q24 (I)<br>T27 (I)	S19 (I)<br>Q24 (I)	S19 (I)<br>Q24 (I)<br>T27 (I)
G476	S19 (I)<br>Q24 (I)	S19 (I)<br>Q24 (I)	I21 (I)<br>Q24 (I)	I21 (I)<br>Q24 (I)	D463	S19 (I)<br>Q24 (I)	S19 (I)	Q24 (H)<br>S19 (I)	S19 (I)	S19 (I)
S477	S19 (I)<br>Q24 (I)	Q24 (I)	Q24 (I)							
E484	K31 (I)	K31 (I)			P470	L79 (I)	E75 (I)	E75 (I)<br>L79 (I)		L79 (I)
G485	L79 (I)	L79 (I)	L79 (I)	L79 (I)	A471			L79 (I)		
F486	Q24 (I)<br>L79 (I)<br>M82 (I)<br>Y83 (I)	Q24 (I)<br>L79 (I)<br>M82 (I)<br>Y83 (I)	Q24 (I)<br>L79 (I)<br>M82 (I)<br>Y83 (I)	Q24 (I)<br>L79 (I)<br>M82 (I)<br>Y83 (I)	L472	L79 (I)<br>M82 (I)<br>Y83 (I)	L79 (I)<br>M82 (I)	L79 (I)<br>M82 (I)<br>Y83 (I)	T78 (I)<br>L79 (I)<br>M82 (I)	F28 (I)<br>L79 (I)<br>M82 (I)<br>Y83 (I)
N487	Q24 (H)<br>A25 (I)<br>T27 (I)<br>F28 (I)<br>Y83 (H)	Q24 (H)<br>T27 (I)<br>F28 (I)<br>L79 (I)<br>Y83 (H)	Q24 (I)<br>T27 (I)<br>F28 (I)<br>M82 (I)<br>Y83 (I)	Q24 (I)<br>T27 (I)<br>F28 (I)<br>M82 (I)<br>Y83 (I)	N473	Q24 (H)<br>Y83 (H)	Q24 (H)<br>M82 (I)<br>Y83 (H)	Q24 (H)<br>A25 (I)<br>T27 (I)<br>F28 (I)<br>Y83 (H)	Q24 (I)<br>F28 (I)<br>L79 (I)<br>M82 (I)<br>Y83 (H)	Q24 (H)<br>A25 (I)<br>T27 (I)<br>F28 (I)<br>Y83 (H)
					C474	K31 (I)	K31 (I)	K31 (I)		
Y489	Q24 (I)<br>T27 (I)<br>F28 (I)<br>L29 (I)<br>K31 (I)<br>Y83 (H)	Q24 (I)<br>T27 (I)<br>F28 (I)<br>K31 (I)<br>Y83 (H)	Q24 (I)<br>T27 (H)<br>F28 (I)<br>L29 (I)<br>K31 (I)<br>Y83 (I)	Q24 (I)<br>T27 (H)<br>F28 (I)<br>K31 (I)<br>Y83 (I)	Y475	Q24 (I)<br>T27 (I)<br>F28 (I)<br>K31 (I)<br>Y83 (H)	Q24 (I)<br>T27 (I)<br>F28 (I)<br>K31 (I)<br>Y83 (H)	Q24 (I)<br>T27 (I)<br>F28 (I)<br>L29 (I)<br>K31 (I)<br>Q76 (I)<br>L79 (I)<br>Y83 (H)	Q24 (H)<br>T27 (I)<br>F28 (I)<br>K31 (I)<br>L79 (I)<br>Y83 (H)	F28 (I)<br>K31 (I)<br>Q76 (I)<br>L79 (I)
F490	K31 (H)	K31 (I)	K31 (I)	K31 (I)	W476			K31 (I)	K31 (I)	

L492	K31 (I)		H34 (I)	
Q493	H34 (I)<br>E35 (H)	K31 (I)<br>H34 (I)<br>E35 (H)<br>A36 (I)<br>D38 (I)	K31 (H)<br>H34 (I)<br>E35 (H)	K31 (H)<br>H34 (I)<br>E35 (H)
S494			H34 (I)<br>D38 (I)	H34 (I)<br>D38 (I)
Y495	D38 (I)<br>K353 (I)		H34 (I)<br>D38 (I)	H34 (I)<br>D38 (I)
G496	D38 (I)<br>K353 (H)	D38 (I)<br>K353 (I)	H34 (I)<br>D38 (I)<br>K353 (H)	H34 (I)<br>D38 (I)<br>K353 (H)
Q498	D38 (I)<br>Y41 (I)<br>Q42 (H)<br>L45 (I)<br>K353 (I)	D38 (I)<br>Y41 (I)<br>Q42 (I)<br>L45 (I)<br>K353 (I)	D38 (I)<br>Y41 (I)<br>Q42 (I)<br>L45 (I)<br>K353 (I)	D38 (I)<br>Y41 (I)<br>Q42 (I)<br>L45 (I)<br>K353 (I)
T500	Y41 (H)<br>L45 (I)<br>G326 (I)<br>N330 (I)<br>K353 (I)<br>G354 (I)<br>D355 (I)<br>R357 (I)	Y41 (H)<br>L45 (I)<br>G326 (I)<br>N330 (I)<br>K353 (I)<br>G354 (I)<br>D355 (I)<br>R357 (I)	Y41 (H)<br>L45 (I)<br>N330 (I)<br>K353 (I)<br>D355 (I)<br>R357 (I)	Y41 (H)<br>L45 (I)<br>N330 (I)<br>K353 (I)<br>D355 (I)<br>R357 (I)
N501	Y41 (H)<br>N330 (I)<br>G352 (I)<br>K353 (I)<br>G354 (I)<br>D355 (I)	Y41 (H)<br>G326 (I)<br>N330 (I)<br>K353 (I)<br>G354 (I)<br>D355 (I)	Y41 (H)<br>K353 (I)<br>G354 (I)<br>D355 (I)	Y41 (H)<br>K353 (I)<br>G354 (I)<br>D355 (I)
G502	Y41 (I)<br>K353 (H)<br>G354 (I)<br>D355 (I)	Y41 (I)<br>K353 (H)<br>G354 (I)<br>D355 (I)	Y41 (I)<br>T324 (I)<br>K353 (H)<br>G354 (I)<br>D355 (I)	Y41 (I)<br>T324 (I)<br>K353 (H)<br>G354 (I)<br>D355 (I)

N479	D30 (I)<br>K31 (I)<br>H34 (I)	D30 (I)<br>K31 (I)<br>H34 (I)<br>E35 (I)	D30 (I)<br>K31 (I)<br>H34 (I)<br>E35 (I)	K31 (I)<br>H34 (I)<br>E35 (I)	K31 (I)<br>H34 (I)<br>E35 (I)
D480	D38 (I)		H34 (I)	H34 (I)<br>D38 (I)	H34 (I)
Y481	D38 (I)<br>K353 (I)	D38 (I)<br>K353 (H)	D38 (I)<br>K353 (I)	H34 (I)<br>E37 (I)<br>D38 (I)<br>K353 (I)	D38 (I)<br>K353 (I)
G482	D38 (I)<br>K353 (I)	D38 (I)<br>K353 (H)	D38 (I)<br>K353 (I)	E37 (I)<br>D38 (I)<br>K353 (I)	D38 (I)<br>Y41 (I)<br>K353 (I)
F483	K353 (I)		K353 (I)	D38 (I)<br>K353 (I)	K353 (I)
Y484	D38 (I)<br>Y41 (I)<br>Q42 (I)<br>L45 (I)<br>K353 (I)	D38 (H)<br>Y41 (I)<br>Q42 (I)<br>L45 (I)<br>K353 (I)	D38 (I)<br>Y41 (I)<br>Q42 (I)<br>L45 (I)<br>K353 (I)	D38 (I)<br>Y41 (I)<br>Q42 (H)<br>L45 (I)<br>K353 (I)	D38 (I)<br>Y41 (I)<br>Q42 (H)<br>L45 (I)<br>K353 (I)
T485	Y41 (I)<br>E329 (I)<br>E330 (I)	Y41 (I)<br>E329 (I)	E329 (I)<br>N330 (I)	E329 (I)<br>N330 (I)	Y41 (I)<br>G326 (I)<br>E329 (I)<br>E330 (I)
T486	Y41 (H)<br>L45 (I)<br>G326 (I)<br>N330 (H)<br>K353 (I)<br>G354 (I)<br>D355 (I)<br>R357 (I)	Y41 (H)<br>L45 (I)<br>G326 (I)<br>E329 (I)<br>N330 (H)<br>K353 (I)<br>G354 (I)<br>D355 (I)<br>R357 (I)	Y41 (H)<br>L45 (I)<br>G326 (I)<br>E329 (I)<br>N330 (H)<br>K353 (I)<br>G354 (I)<br>D355 (I)<br>R357 (I)	Y41 (H)<br>L45 (I)<br>G326 (I)<br>N330 (H)<br>K353 (H)<br>G354 (I)<br>D355 (H)<br>R357 (I)	Y41 (H)<br>L45 (I)<br>T324 (I)<br>Q325 (I)<br>G326 (I)<br>E329 (I)<br>N330 (H)<br>G354 (I)<br>D355 (I)<br>R357 (H)
T487	Y41 (H)<br>G326 (I)<br>N330 (I)<br>K353 (I)<br>G354 (I)<br>D355 (I)<br>R357 (I)	Y41 (H)<br>G326 (I)<br>N330 (I)<br>K353 (H)<br>G354 (I)<br>D355 (I)	Y41 (I)<br>E329 (I)<br>N330 (I)<br>K353 (I)	Y41 (I)<br>Q325 (I)<br>G326 (I)<br>E329 (I)<br>N330 (I)<br>K353 (I)<br>G354 (I)<br>D355 (I)	Y41 (I)<br>T324 (I)<br>Q325 (I)<br>G326 (I)<br>E329 (I)<br>N330 (I)<br>K353 (I)<br>G354 (I)<br>D355 (I)<br>R357 (I)
G488	Y41 (I)<br>T324 (I)<br>G352 (I)<br>K353 (H)<br>G354 (I)<br>D355 (I)	Y41 (I)<br>T324 (I)<br>K353 (H)<br>G354 (I)<br>D355 (I)	G326 (I)<br>K353 (H)<br>G354 (I)<br>D355 (I)	T324 (I)<br>Q325 (I)<br>G326 (I)<br>N330 (I)<br>K353 (H)<br>G354 (I)	T324 (I)<br>Q325 (I)<br>G326 (I)<br>N330 (I)<br>K353 (H)<br>G354 (I)

									D355 (I)	D355 (I)
V503	Q325 (I)<br>K353 (I)<br>G354 (I)	Q325 (I)<br>K353 (I)<br>G354 (I)	T324 (I)<br>G326 (I)<br>K353 (I)<br>G354 (I)	T324 (I)<br>G326 (I)<br>K353 (I)<br>G354 (I)	I489	Q325 (I)<br>G326 (I)<br>K353 (I)<br>G354 (I)	Q325 (I)<br>G354 (I)	T324 (I)<br>Q325 (I)<br>G326 (I)<br>G354 (I)	T324 (I)<br>Q325 (I)<br>G326 (I)<br>G354 (I)	T324 (I)<br>Q325 (I)<br>G326 (I)
**Y505**	E37 (H)<br>K353 (I)<br>G354 (I)<br>A386 (I)<br>R393 (I)	E37 (H)<br>K353 (I)<br>G354 (I)<br>A386 (I)<br>R393 (H)	E37 (H)<br>K353 (I)<br>G354 (I)<br>A386 (I)<br>R393 (I)	E37 (H)<br>K353 (I)<br>G354 (I)<br>A386 (I)<br>R393 (I)	**Y491**	E37 (H)<br>K353 (I)<br>G354 (I)<br>D355 (I)<br>R393 (I)	E37 (H)<br>K353 (I)<br>G354 (I)<br>D355 (I)<br>A386 (I)<br>R393 (I)	K353 (I)<br>G354 (I)<br>D355 (I)<br>A386 (I)<br>R393 (H)	E37 (I)<br>K353 (I)<br>G354 (I)<br>R393 (I)	K353 (I)<br>G354 (I)<br>R393 (I)
					Q492	Q325 (I)<br>G326 (I)<br>E329 (I)	Q325 (I)	Q325 (I)<br>E329 (I)	Q325 (I)<br>E329 (I)	Q325 (I)<br>E329 (I)
