# Supplementary figures and images for: Structural Dissection of Viral Spike-Protein Binding of SARS-CoV-2 and SARS-CoV-1 to the Human Angiotensin-Converting Enzyme 2 (ACE2) as Cellular Receptor
Source: Biomedicines. 2021 Aug 18;9(8):1038. doi: 10.3390/biomedicines9081038 (PMC8394803; doi:10.3390/biomedicines9081038)

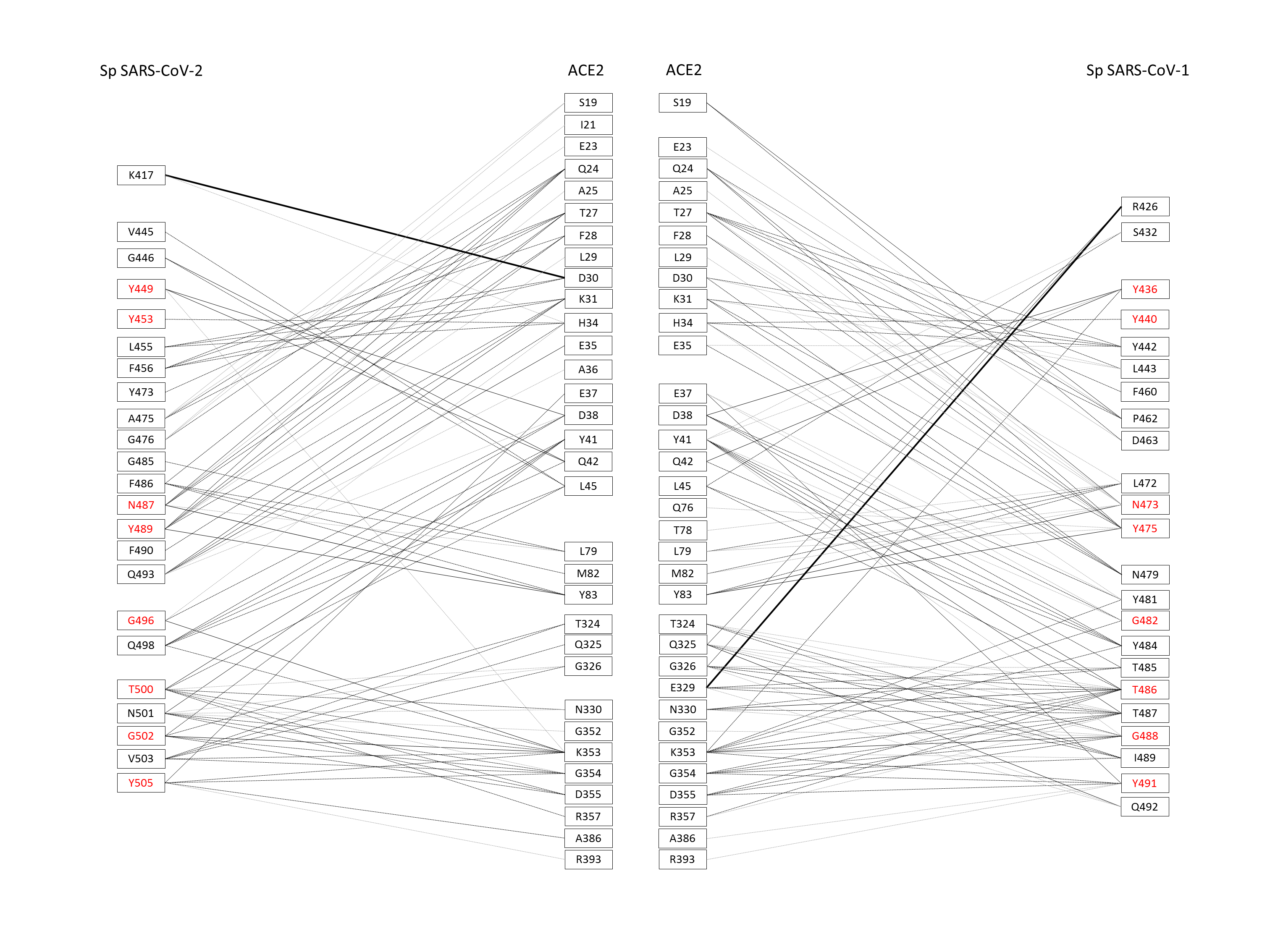

Supplement: Supplementary file 1 [file biomedicines-09-01038-s001.zip › Figure-S1.png]
